# Anthocyanin Synthesis Capability of Maize Cultivars Is Associated with Their Photosynthetic Carbon Partitioning for Growth Adaptability Under Low Phosphorus

**DOI:** 10.3390/plants14172690

**Published:** 2025-08-28

**Authors:** Wang Tang, Zu-Dong Xiao, Xing-Wei Liang, Si Shen, Xiao-Gui Liang, Shun-Li Zhou

**Affiliations:** 1 State Key Laboratory of Maize Bio-Breeding, College of Agronomy and Biotechnology, China Agricultural University, Beijing 100193, China; tw2089203599@163.com (W.T.);; 2 Ministry of Education Key Laboratory of Crop Physiology, Ecology and Genetic Breeding, Jiangxi Agricultural University, Nanchang 330045, China; 3 Innovation Center of Agricultural Technology for Lowland Plain of Hebei, Wuqiao 061802, China

**Keywords:** anthocyanin, maize cultivars, carbohydrate, photosynthetic carbon partitioning, low phosphorus

## Abstract

Anthocyanins (ACNs) are flavonoid pigments that accumulate in plants and respond to environmental stimuli, including low phosphorus (LP). The synthesis and stable accumulation of ACNs rely on substantial carbohydrate investment, implying a potential role in carbon partitioning-mediated growth and resistance, in addition to the well-established antioxidant activity. To investigate cultivar-dependent differences in ACN accumulation and their relationship with photo-assimilate partitioning and growth adaptation under LP stress, seedlings of six representative maize cultivars were hydroponically cultured under both control and LP conditions. ACNs content, photosynthetic parameters, plant relative growth ratio, and tissue-specific carbohydrates were quantified. The results showed that LP reduced photosynthesis and biomass, while stimulating ACNs biosynthesis in leaves and sheaths. Cultivars were then classified as ACN-sensitive and -insensitive groups based on the ACNs accumulation in the newly unfolded leaves and corresponding sheaths. ACN-sensitive cultivars exhibited higher ACNs levels, which correlated positively with soluble sugars but negatively with starch reserves, suggesting preferential carbon partitioning to ACNs precursors rather than to starch. These cultivars also maintained higher relative growth ratios under LP, associated with less photosynthesis decline and starch accumulation compared with ACN-insensitive cultivars. We hypothesize that ACNs synthesis function as a diversion of photo-assimilates into secondary metabolism under LP, thereby improving photosynthetic efficiency by mitigating excess sugar accumulation that could impair plant growth. This carbon-partitioning adaptation could be exploited by selecting for ACNs accumulation as a breeding trait to enhance maize resilience to LP.

## 1. Introduction

Anthocyanins (ACNs) are a group of flavonoids widely distributed in plants and have been developed as nutraceuticals and pharmaceuticals because of their strong antioxidant activity [1]. As secondary metabolites, ACNs are typically found in leaves, seeds, and flowers, where they play crucial roles in physiological processes and environmental responses, including inhibiting oxidases, eliminating ROS, and activating antioxidant enzymes, particularly under stress conditions such as low phosphorus (LP) or low temperature [2]. For example, enhanced ACNs accumulation through overexpression of UDP-glycosyltransferase improved plant tolerance to cold, salinity, and drought stress [3]. With growing interest in ACNs and other flavonoids, numerous studies have focused on elucidating their biosynthetic pathways, transcriptional regulation, biological activities, and underlying mechanisms in flowers, fruits, and vegetables [4,5,6]. In cereal crops such as maize, ACNs also accumulate in specific tissues, influenced by environmental stimuli and varying among cultivars [7]. However, these differences and their implications have not been fully investigated. Understanding these differences, particularly cultivars’ responses to environmental stimuli, is essential for improving crop growth and resilience.

Maize (*Zea mays* L.) is an important global food crop, in which ACNs are among the predominant flavonoid pigments present in tissues such as leaves, sheaths, and silk [8,9]. In these tissues, ACNs typically impart red or purple coloration. Chemically, ACNs are derived from anthocyanidins, which are stabilized through glycosylation in the presence of sugars [10]. Approximately 30–40% of photo-assimilates are estimated to be partitioned to the phenylpropanoid pathway, in which ACN accumulation is a major process, particularly under environmental stimuli [11,12]. Hence, ACN accumulation requires carbohydrate consumption and may divert photosynthetically fixed carbon into secondary metabolism. Moreover, sugars act as key regulators of gene expression involved in ACNs biosynthesis [10,13]. Numerous studies have shown that the ACNs content in plants closely reflect the local accumulation of non-structural carbohydrates, including sucrose, glucose, fructose, and in some cases, starch. Exogenous application of sugars, particularly sucrose, significantly promotes ACNs synthesis and induces the expression of related genes [14,15,16]. In maize, carbohydrates synthesized in leaves through photosynthesis are temporarily stored as total soluble carbohydrates (TSC) and starch, or transported via phloem vascular bundles in the leaf and sheath [17]. Interestingly, ACNs primarily accumulate in epidermal cells adjacent to vascular bundles, further implying a strong link between ACNs and sugars [18]. As an important indicator for cultivar identification, ACN accumulation varies among cultivars and in response to environmental factors, potentially reflecting changes in sugar distribution and utilization within plants. However, the relationship between cultivar-specific ACNs response to environmental stimuli and the associated changes in tissue-specific sugar metabolism is understudied.

Phosphorus (P) is an essential macronutrient for maize, functioning in molecular structure, protein activity, energy metabolism, and signal transduction [19]. P deficiency inhibits photosynthesis, reduces maize growth, and alters the root/shoot ratio by modifying carbon allocation for P acquisition [20,21]. Consistent with low temperature and ultraviolet stress, the accumulation of ACNs in maize can be stimulated by LP, and the underlying physiological mechanisms are relatively well characterized [22]. Nevertheless, how ACN accumulation influences plant adaptation to LP remains poorly understood, particularly across different maize cultivars. Responses of plant carbon fixation, sugar metabolism, and growth to LP stress differ among cultivars. Furthermore, LP-induced ACNs accumulation may vary within cultivars. Given the sugar dependence of LP-induced ACNs synthesis, this pathway may redirect carbohydrate partitioning and thereby influence plant growth and stress responses. Therefore, investigating the potential linkages between ACNs accumulation, carbon metabolism, and growth adaptation under stresses such as LP is of considerable importance.

In this study, we examined six representative maize cultivars that differ in their capacity for ACNs accumulation. We imposed P deficiency on maize seedlings and investigated the response of ACNs synthesis, leaf photosynthesis, diurnal carbohydrates in different tissues, as well as plant growth, with the aims of (1) clarifying the differences in LP-induced ACNs synthesis among maize cultivars, and (2) exploring the relationships among ACNs accumulation, carbon metabolism, and plant growth. The results will provide insights into how ACN accumulation as a cultivar-specific trait affects plant carbon partitioning, growth, and resilience, and may serve as a potential phenotypic indicator for future breeding.

## 2. Materials and Methods

### 2.1. Plant Material and Treatment

Six widely cultivated commercial maize cultivars, including Denghai605 (DH605), Jingnongke728 (JNK728), Xianyu335 (XY335), Zhengdan958 (ZD958), Weike702 (WK702), and Nongda108 (ND108), representing the majority in the China Corn Belt, were used in this study. The leaf sheath of seedlings is purple for DH605, JNK728, and XY335, but is green for ZD958, WK702, and ND108, according to the certification information, indicating different characteristics of ACNs accumulation. The experiments were conducted in a walk-in growth chamber with a 14/10 h light/dark (L/D) cycle. The artificial light intensity was 250 ± 50 μmol m^−2^ s^−1^, and the air temperatures were 27/25 °C (L/D). Plants were cultivated in nutrient solutions with two phosphorus concentrations: control (CK, *p* = 6.25 × 10^−5^ mol L^−1^) and low phosphorus (LP, *p* = 2.5 × 10^−6^ mol L^−1^), as described previously [23] (Table S1).

### 2.2. Experimental Design

Maize seeds were selected and placed in trays with adequate water supply for germination (BBCH11). Seedlings were transferred after the V1 stage (BBCH11) to plastic pots containing 10 L of 1/2 concentrated complete nutrient solution for 3 days before being transferred to 100% complete nutrient solution with roots continuously aerated using an air pump. Each pot contained 12 plants. At the V2 stage (BBCH 12), each cultivar was subjected to the P treatments (CK and LP) for 9 days. By the end of the treatment, plants were at the V4 stage (BBCH 14). Plants were sampled and separated into different parts, including the uppermost unfolded leaf (the 4th leaf), the corresponding sheath, plant root, new leaves, and the rest. Samples were quickly frozen in liquid nitrogen and stored at −80 °C for further analysis [21].

### 2.3. Plant Growth Parameters

Representative maize seedlings of each cultivar were collected on the days that treatment began (b) and ended (e) to determine plant shoot and root fresh weight (FW), leaf area, and relative growth ratio (RGR). Leaf area was determined as follows: Leaf area (LA) = Leaf length × Maximum leaf width × 0.75 (for unfolded leaves) or ×0.5 (for folded leaves). RGR was calculated as follows: RGR (%) = (ln(FW_e_) − ln(FW_b_))/9 × 100, where “FW_b_” and “FW_e_” indicate fresh weight at the beginning (V2, BBCH12) and end (V4, BBCH14) of the 9-day LP treatment, respectively. Five replicates were measured.

### 2.4. Leaf Photosynthesis Rate

The net photosynthetic rate of the 4th leaf was determined using a LI-6400 system (LI-COR Inc., Lincoln, NE, USA) for each cultivar with three biological replicates. Measurements were conducted in the first half of the light period (8:30–14:00) on the final day of LP treatment under control conditions: CO_2_ concentration, 400 μmol mol^–1^; photosynthetic photon flux density (PPFD), 250 μmol m^–2^ s^–1^; block temperature, 25 °C; and flow setting, 200 μmol s^–1^ following Liang et al. [24].

### 2.5. Carbohydrates and Anthocyanins

Carbohydrates, including sucrose, total soluble carbohydrates (TSC), and starch were determined on the morning (end of the night, 8:00) and evening (end of the day, 22:00) of the last day of treatment for the 4th leaf, the corresponding sheath, roots, and unfolded leaves, each with 5 bioduplicates. Starch and TSC were determined using the anthrone–sulfuric acid method, and sucrose was measured with a modified resorcinol method [25,26]. Briefly, samples were ground in liquid nitrogen, and ~100 mg was weighed into centrifuge tubes. Samples were extracted with 1 mL of 80% (*v*/*v*) ethanol, incubated in an 80 °C water bath for 30 min, shaken, and centrifuged. The extraction was repeated twice. Supernatants were combined and decolorized with activated charcoal for sucrose and TSC assay. Starch in the pellet was enzymatically digested into glucose using amyloglucosidase and α-amylase by incubation in a 60 °C water bath for 2 h. Starch and TSC were quantified as glucose equivalents using a spectrophotometer (TU-1901, PRESEE, Beijing, China) at 625 nm, while sucrose was measured at 480 nm.

For ACNs, ~100 mg of the 4th leaf and corresponding sheath were weighed into centrifuge tubes, with five replicates for each. Samples were extracted with 300 μL of 1% HCl (*v*/*v*) in methanol and incubated overnight at 4 °C. Then, 200 μL of distilled water and 500 μL of chloroform were added, followed by centrifugation. Supernatants were collected, and absorbance was measured at 530 and 657 nm to determine ACNs using the following equation: An = [A530–A657]/g FW [27].

### 2.6. Statistical Analysis

Data were initially processed using Excel 2019. SPSS 25.0 was used for ANOVA, Origin 2021pro for histograms, and the “Correlation Plot” plug-in for correlation coefficient matrices. Adobe Illustrator CS6 was used for the schematic diagram, in which our maize seedling was cartoonized using Cici2025 software.

## 3. Results

### 3.1. The Difference in Anthocyanin Accumulation Within Cultivars

**Figure 1:**
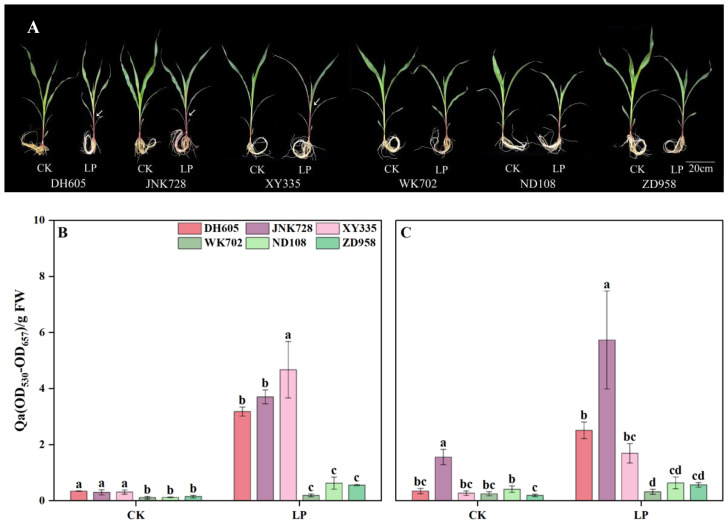
Plant phenotype (**A**) and ACNs accumulation in the 4th leaf (**B**) and its sheath (**C**) of ACN-sensitive cultivars (DH605, JNK728, and XY335) and ACN-insensitive cultivars (WK702, ND108, and ZD958) under control (CK) and low phosphorus (LP) treatments at the V4 stage. The values followed by different letters indicate significant differences at the 0.05 level. The error bars indicate the standard deviation (n = 5). Abbreviations: ACN: anthocyanin; DH605: Denghai605; JNK728: Jingnong728; XY335: Xianyu335; WK702: Weike702; ND108: Nongda108; ZD958: Zhengdan958.

Compared with CK, LP obviously induced ACNs accumulation in the 4th leaf and its sheath across all cultivars (Figure 1). Under CK, the ACNs content ranged from 0.11 to 0.34 in leaves and from 0.19 to 1.56 in sheaths. However, under LP, these values dramatically increased to the range of 0.19 to 4.67 in leaves and 0.32 to 5.73 in sheaths (Figure 1B,C). Notably, the response of ACNs accumulation to LP differed among cultivars. Cluster analysis classified the cultivars into two groups (Figure S1). Sensitive cultivars, including DH605, JNK728, and XY335, exhibited higher ACNs accumulation under both control and LP conditions. In contrast, ACN-insensitive cultivars, including WK702, ND108, and ZD958, showed lower ACNs accumulation under CK and weaker responses to LP stress. Overall, LP-induced ACNs accumulation in ACN-insensitive cultivars was increased by only 9.4% in leaves and 8.6% in sheaths compared with sensitive ones.

### 3.2. The Response of Plant Growth to P Deficiency

**Figure 2:**
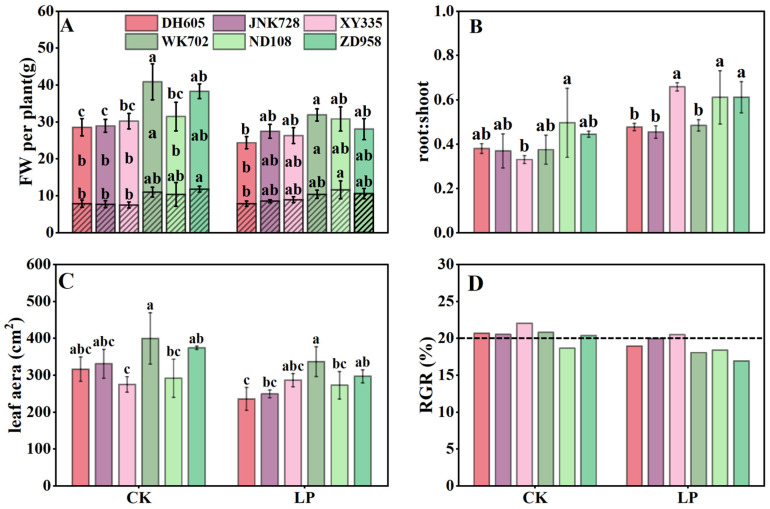
Plant growth parameters of ACN-sensitive and -insensitive cultivars under control (CK) and low phosphorus (LP) treatments at the V4 stage. Fresh weight (FW) of shoots (solid-color-filled columns) and roots (hatched columns) (**A**). Root–shoot ratio (**B**). Leaf area (**C**). Relative growth ratio (RGR); the dashed line means RGR = 20% (**D**). The values followed by different letters indicate significant differences at the 0.05 level. The error bars indicate the standard deviation (n = 4–5). Abbreviations: ACN: anthocyanin; DH605: Denghai605; JNK728: Jingnong728; XY335: Xianyu335; WK702: Weike702; ND108: Nongda108; ZD958: Zhengdan958.

Under CK, the overall fresh weight of ACN-insensitive cultivars (WK702, ND108, and ZD958) was higher than that of the sensitive ones, which was mainly due to greater root biomass, as reflected by the root-to-shoot ratio (Figure 2A,B). Although leaf area and RGR differed among hybrids, no obvious differences were detected between the two ACN types (Figure 2C,D). LP treatment decreased plant fresh weight, leaf area, and RGR, but increased the root-to-shoot ratio. The FW of ACN-insensitive cultivars remained generally higher than that of sensitive ones, but the root biomass was comparable, resulting in no significant difference in root-to-shoot ratio between the two types (Figure 2A,B). Generally, the ACN-sensitive cultivars exhibited a smaller RGR reduction (6.1%) compared with ACN-insensitive ones (10.7%) (Figure 2D).

### 3.3. Leaf Photosynthesis Affected by LP

**Figure 3:**
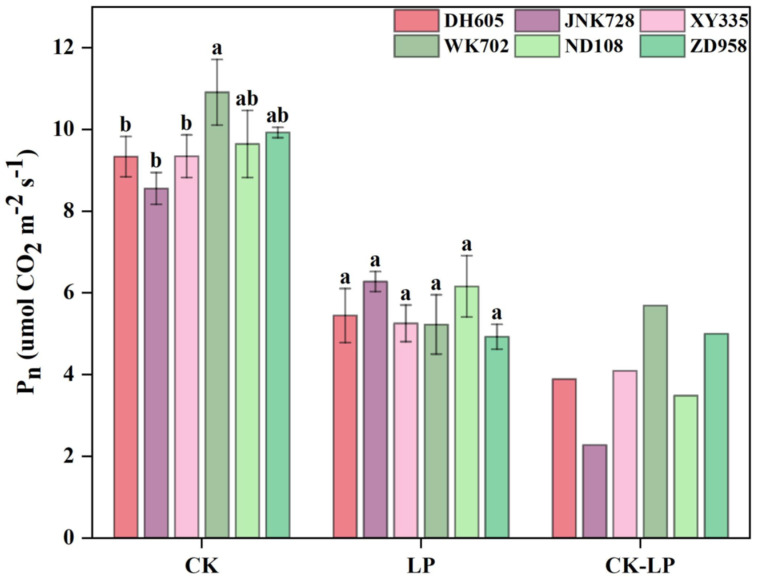
Photosynthesis of ACN-sensitive and -insensitive cultivars in the 4th leaf under control (CK) and low phosphorus (LP) treatments, and the difference between them (CK-LP) at the V4 stage. The values followed by different letters indicate significant differences at the 0.05 level. The error bars indicate the standard deviation (n = 3). Abbreviations: ACN: anthocyanin; DH605: Denghai605; JNK728: Jingnong728; XY335: Xianyu335; WK702: Weike702; ND108: Nongda108; ZD958: Zhengdan958.

Under CK, net photosynthesis of the fourth leaf differed among the six cultivars (Figure 3). Compared with ACN-insensitive cultivars (WK702, ND108, and ZD958), the photosynthetic rates were slightly or significantly lower for the ACN-sensitive ones (DH605, JNK728, and XY335). Under LP conditions, photosynthetic rates of all cultivars decreased substantially by 30–40% (Figure 3). Notably, the significant difference in photosynthesis between the two groups disappeared, suggesting that the LP-induced reduction in photosynthetic rate was partially alleviated in ACN-sensitive cultivars.

### 3.4. Carbohydrate Accumulation in the Fourth Leaf, Sheaths, Unexpanded New Leaves, and Roots

**Figure 4:**
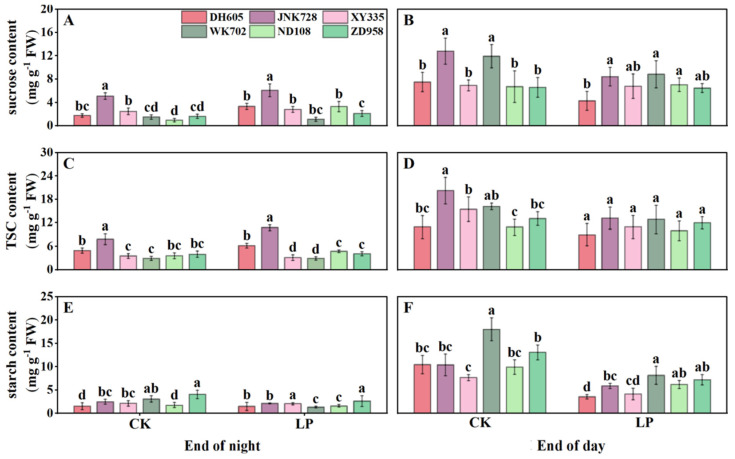
Diurnal carbohydrate levels of ACN-sensitive and -insensitive cultivars in the 4th leaf (main source leaf) under control (CK) and low phosphorus (LP) treatments at the V4 stage. Contents of sucrose (**A**,**B**), total soluble carbohydrates (TSC) (**C**,**D**), and starch (**E**,**F**) at the end of the night (8:00) and the end of the day (22:00), respectively. The values followed by different letters indicate significant differences at the 0.05 level. The error bars indicate the standard deviation (n = 5). Abbreviations: ACN: anthocyanin; DH605: Denghai605; JNK728: Jingnong728; XY335: Xianyu335; WK702: Weike702; ND108: Nongda108; ZD958: Zhengdan958.

At the end of the night, sucrose and TSC in the 4th leaf were higher in ACN-sensitive cultivars (DH605, JNK728, and XY335) than in ACN-insensitive ones (WK702, ND108, and ZD958), whereas starch content did not differ between the two types (Figure 4A,C,E). Values under LP were comparable to those under CK. By contrast, at the end of the day, sugar content differed from that observed at the end of the night. Although sucrose and TSC levels did not differ between cultivar groups, the starch content was generally lower in cultivars with higher ACNs accumulation. Values under LP were again comparable to CK (Figure 4B,D,F). Overall, LP tended to reduce sugar accumulation, including both TSC and starch, in newly expanded leaves during the daytime. Additionally, starch accumulation was lower in ACN-sensitive cultivars than in ACN-insensitive ones (Figure 4E,F).

**Figure 5:**
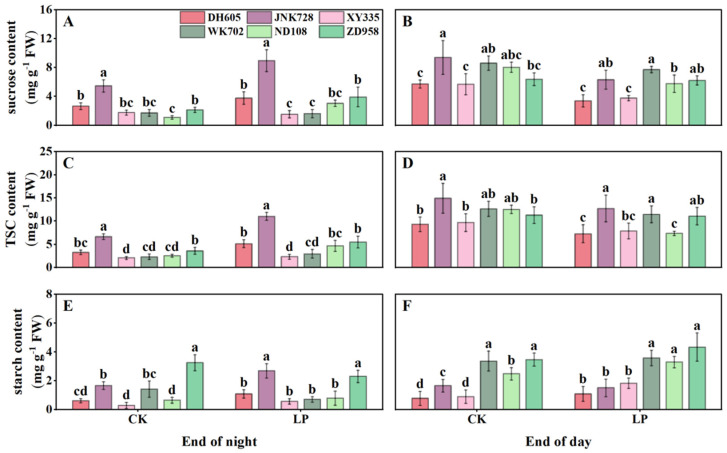
Diurnal carbohydrate levels of ACN-sensitive and -insensitive cultivars in the sheaths of the 4th leaf under control (CK) and low phosphorus (LP) treatments at the V4 stage. Contents of sucrose (**A**,**B**), total soluble carbohydrates (TSC) (**C**,**D**), and starch (**E**,**F**) at the end of the night (8:00) and the end of the day (22:00), respectively. The values followed by different letters indicate significant differences at the 0.05 level. The error bars indicate the standard deviation (n = 5). Abbreviations: ACN: anthocyanin; DH605: Denghai605; JNK728: Jingnong728; XY335: Xianyu335; WK702: Weike702; ND108: Nongda108; ZD958: Zhengdan958.

In the sheath of the fourth leaf, sucrose, TSC, and starch levels varied among cultivars but were not affected by ACNs sensitivity at the end of the night (Figure 5A,C,E). Generally, sugar levels were higher at the end of the day than at night, but sucrose and TSC levels were similar between the two groups (Figure 5B,D,F). Notably, daytime starch accumulation was consistently lower in ACN-sensitive cultivars than in ACN-insensitive ones, under both CK and LP conditions (Figure 5F).

**Figure 6:**
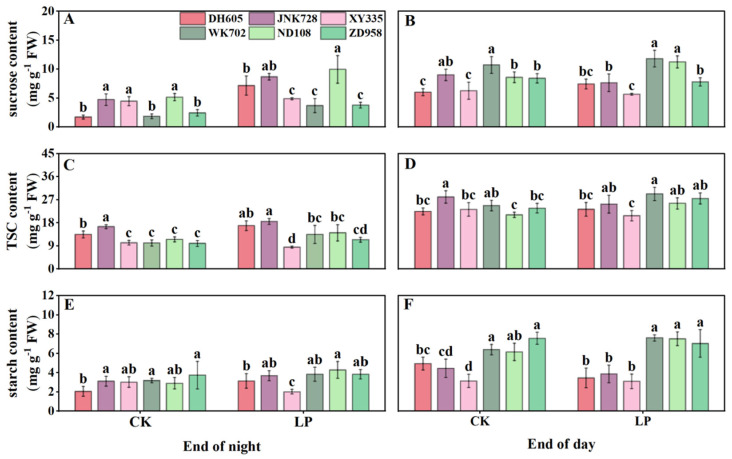
Diurnal carbohydrate levels of ACN-sensitive and -insensitive cultivars in the unexpanded new leaves under control (CK) and low phosphorus (LP) treatments at the V4 stage. Contents of sucrose (**A**,**B**), total soluble carbohydrates (TSC) (**C**,**D**), and starch (**E**,**F**) at the end of the night (8:00) and the end of the day (22:00), respectively. The values followed by different letters indicate significant differences at the 0.05 level. The error bars indicate the standard deviation (n = 5). Abbreviations: ACN: anthocyanin; DH605: Denghai605; JNK728: Jingnong728; XY335: Xianyu335; WK702: Weike702; ND108: Nongda108; ZD958: Zhengdan958.

In unexpanded new leaves, LP treatment significantly increased sucrose content in all cultivars at the end of the night, but TSC and starch did not differ significantly, nor were there variations in the starch content between cultivar groups at the end of the night (Figure 6A,C,E). By contrast, under LP at the end of the day, sugars, particularly sucrose, and starch contents were significantly lower in ACN-sensitive cultivars than in ACN-insensitive ones (Figure 6B,D,F).

**Figure 7:**
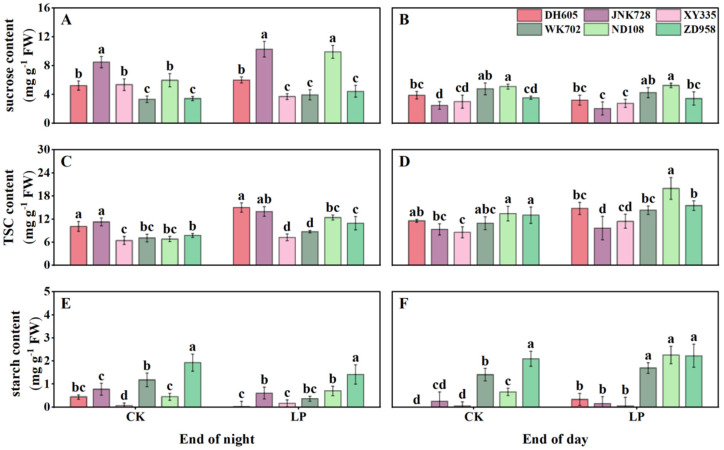
Diurnal carbohydrate levels of ACN-sensitive and -insensitive cultivars in the roots under control (CK) and low phosphorus (LP) treatments at the V4 stage. Contents of sucrose (**A**,**B**), total soluble carbohydrates (TSC) (**C**,**D**), and starch (**E**,**F**) at the end of the night (8:00) and the end of the day (22:00), respectively. The values followed by different letters indicate significant differences at the 0.05 level. The error bars indicate the standard deviation (n = 5). Abbreviations: ACN: anthocyanin; DH605: Denghai605; JNK728: Jingnong728; XY335: Xianyu335; WK702: Weike702; ND108: Nongda108; ZD958: Zhengdan958.

In roots, diurnal sugar accumulation during the daytime was not observed (Figure 7). P deficiency slightly increased root sucrose at the end of the night, but TSC content increased significantly, suggesting elevated glucose and fructose levels, with TSC content also rising at the end of the day (Figure 7). At the end of the night, sugar levels, including sucrose, TSC, and starch, were similar between the two cultivar groups (Figure 7A,C,E). Notably, similar to the observations in leaves and sheaths, the starch content at the end of the day was significantly lower in ACN-sensitive cultivars than in ACN-insensitive ones (Figure 7B,D,F).

### 3.5. Correlations Among Anthocyanins, Plant Growth, Photosynthesis, and Tissue-Specific Carbohydrates

**Figure 8:**
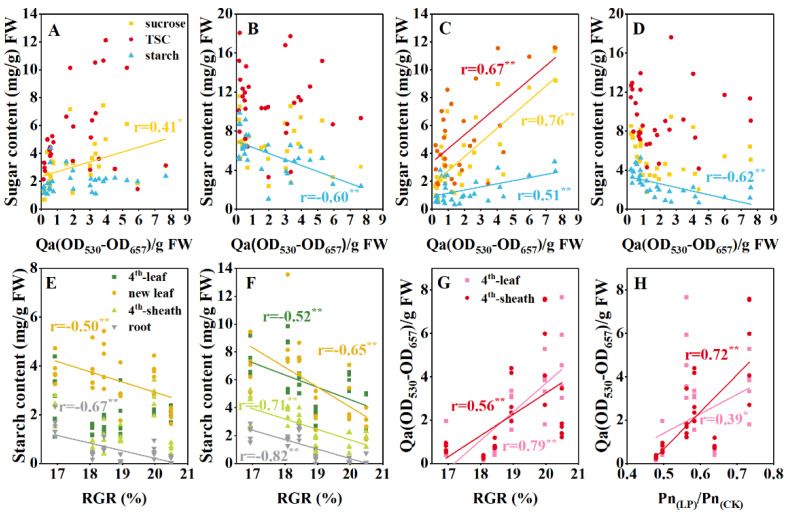
Correlation coefficient of six cultivars under low phosphorus (LP) at the V4 stage. The correlation between ACNs and carbohydrate content in the 4th leaf at the end of the night (**A**) and at the end of the day (**B**), and in the sheaths at the end of the night (**C**) and at the end of day (**D**). The correlation between starch content in the 4th leaf, sheaths, unexpanded new leaves, and roots with RGR at the end of the night (**E**) and at the end of the day (**F**). The correlation between ACNs content and RGR (**G**). The maintenance ratio of photosynthesis (**H**). * and ** indicate significant differences at the 0.05 and 0.01 levels, respectively. Abbreviations: ACN: anthocyanin; RGR: relative growth ratio.

To investigate the relationships between ACNs, sugars, and plant growth under CK and LP conditions, we conducted a correlation analysis on ACNs accumulation in leaves and sheaths, tissue-specific carbohydrates, and growth parameters across the six cultivars. Under CK, ACNs in both the leaves and sheaths were significantly positively correlated with sucrose and TSC at the end of the night. Conversely, at the end of the day, ACNs in leaves were negatively correlated with starch content across tissues, whereas ACNs in sheaths were positively correlated with TSC in the shoot tissues. ACNs accumulation in leaves were negatively correlated with the photosynthetic rate but positively correlated with RGR (Figure S2). Under LP treatment, ACNs in leaves were significantly positively correlated with sucrose, and ACNs accumulation in sheaths were also significantly positively correlated with measured carbohydrates at the end of the day. In contrast, the ACNs content in both leaves and sheaths was significantly negatively correlated with starch levels at the end of the night, with no correlations detected with soluble sugars (Figure S3; Figure 8A–D). Starch contents across the tissues in the daytime were consistently negatively correlated with RGR, slightly or significantly (Figure S3; Figure 8E,F). Importantly, the ACNs content in both leaves and sheaths was found to positively correlate with RGR and the maintenance of photosynthesis (Figure 8G,H).

## 4. Discussion

### 4.1. The Association Between ACNs Accumulation and Carbon Partitioning for Different Cultivar Groups

**Figure 9:**
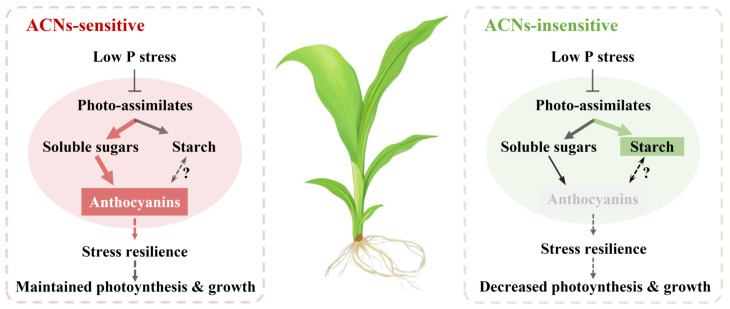
Schematic representation of plant carbon partitioning in ACN-sensitive and -insensitive maize cultivars under low phosphorus stress. Arrows indicate promotion or inhibition, and line thickness indicates preferred photo-assimilates partitioning. Abbreviation: ACN: anthocyanin.

Phosphorus deficiency inhibits photosynthesis and biomass accumulation [21]. In response, newly fixed carbon is preferentially allocated to roots to increase phosphorus uptake [28] (Figure 2). Although ACNs are regulated by sugar signaling and require substantial sugars for biosynthesis, their feedback effects on plant carbon partitioning have received little attention. Here, by comparing ACN-sensitive and -insensitive cultivars under normal and LP conditions, we showed significant differences in diurnal sugar partitioning in mature leaves, sheaths, unexpanded leaves, and roots between the two cultivar groups (Figure 4, Figure 5, Figure 6 and Figure 7). Generally, ACNs accumulation in both leaves and sheaths were positively correlated with sucrose and TSC at the end of the night, although these correlations were weaker by the end of the day (Figure 8, Figures S2 and S3). The close correlation between ACNs synthesis and soluble sugars has been demonstrated many times for reasons mentioned above [14,15,16]. Starch, however, has been less reported to be related to ACNs accumulation. Here, we found that the starch content was consistently lower in ACN-sensitive cultivars than in ACN-insensitive ones across all tissues (Figure 4, Figure 5, Figure 6 and Figure 7). Notably, significant negative correlations were observed between ACNs accumulation and starch reserves across all tissues under both control and LP conditions (Figure 4, Figure 5, Figure 6, Figure 7 and Figure 8). Thus, for the first time, we demonstrated a possible trade-off between ACNs and starch accumulation in different maize cultivars. Normally, plant photo-assimilates are partitioned into sugars or sugar-derived secondary metabolites [24,29,30]. Considering that both the sucrose and TSC levels in leaves and sheaths were rather comparable between the two cultivar groups, and were even lower in new leaves and root for ACN-sensitive cultivars than in ACN-insensitive ones under LP, we speculated that ACN-sensitive cultivars may preferentially redirect newly fixed photo-assimilates towards biosynthesis of ACNs and other secondary metabolites rather than starch storage during daytime (Figure 9). Mechanistically, this metabolic preference may be influenced by the direct regulation of genes, enzymes, and/or transcription factors involved in ACNs biosynthesis with light stimuli [31]. Additionally, enzymatic activities related to sugar metabolism, such as sucrose synthase, and AGPase, may shift carbon flux from starch to soluble sugars, thereby providing substrates for ACNs synthesis. Sugars have been shown to regulate ACNs biosynthesis, either independently or through interactions with hormones [32]. However, further research is required to elucidate the regulatory differences in ACNs synthesis among maize cultivars, especially the core control points of sugar metabolism.

### 4.2. Capability of ACNs Accumulation Is Associated with Photosynthesis and Growth for Different Cultivar Groups Under LP

Previous studies have shown that ACNs accumulation enhances plant resistance to adverse environmental conditions by protecting the photosynthetic apparatus and mitigating cytotoxicity caused by oxidative damage [33]. For example, foliar application of ACNs to maize leaves under LP improved photosynthesis and alleviated biomass loss [34]. However, the connections between ACNs biosynthesis (or even secondary metabolism) and sugar demands, which are fundamental to both growth and stress resistance, have not been thoroughly addressed in relation to plant environmental responses. Here, we found that ACNs accumulation conferred benefits in both preserving photosynthesis and sustaining growth under LP (Figure 2 and Figure 3). Despite photosynthetic rates decreasing across all cultivars under LP (Figure 3), ACN-sensitive cultivars exhibited an average decline that was 8.85% smaller than that of ACN-insensitive ones (Figure 3). This resilience was positively correlated with ACNs content across the six cultivars, suggesting a protective role of ACNs against LP stress (Figure 8). This mitigation likely involves crosstalk between ACNs biosynthesis and sugar metabolism. Inappropriate starch accumulation has been considered detrimental to plant growth [28,29,35]. This is consistent with the negative correlations observed between RGR and starch reserves under LP (Figure 8). ACNs accumulation may serve as an alternative sink for sugars, potentially alleviating the negative effects of starch accumulation on photosynthesis and growth under LP. In contrast, under sufficient nutrition conditions, ACNs accumulation, primarily in the epidermal cells of sensitive cultivars, may reduce light incidence and thereby limit growth, as reflected in photosynthesis and FW under CK (Figure 2 and Figure 3). Hence, ACNs biosynthesis may reflect a balance of carbon partitioning between stress defense and growth, with sensitive cultivars that accumulate ACNs achieving a “stress resilience–growth trade-off” that favors survival under LP (as illustrated in Figure 9).

### 4.3. The Stability of ACNs Synthesis Among Cultivars May Provide a Diagnostic Indicator for Growth or Resistance at an Early Stage

The biosynthesis of ACNs in maize leaves and sheaths exemplifies the typical flavonoid coloration found in different organizations and represents a characteristic trait for the identification and approval of maize cultivars. The genetic basis of flavonoid biosynthesis consists generally of two subgroups: (1) early genes such as *ZmPAL*, *ZmC4H*, and *ZmCHS* that participate in the general phenylpropanoid pathway, providing flavonoid intermediates; (2) later genes such as *a1*, *a2*, *bz1*, *bz2*, and *c2*, engaging in the biosynthesis of specific flavonoids in different tissues [13]. MYB-type transcription factors are key regulators of flavonoid synthesis, controlling both early genes and exerting broad regulatory effects on multiple later-stage genes [36]. For example, the extensively studied R2R3-MYB genes *c1* and *pl1* regulate all the aforementioned later genes, thereby controlling flavonoid accumulation in sheaths, pericarp, husks, culms, cobs, and anther glumes [37,38]. Moreover, MYB gene activity could be stimulated by various environmental stimuli such as low temperature, low phosphorus, and ultraviolet light [39]. Based on these findings, we propose two hypotheses. First, for different maize cultivars, the flavonoid coloration of each organization may be relatively consistent at different growth stages and under different environmental stimuli, as evidenced by the consistency of ACNs accumulation in different types of cultivars under normal and low phosphorus conditions in this study (Figure 1). Second, tissue coloration response to environmental stimuli may be similar due to shared genetic background and common regulation by MYB transcription factors. For instance, a gene linkage between purple sheaths and red silks has been demonstrated in maize double-haploid lines [40]. This suggests that ACNs coloration could serve as an early indicator for yield, grain quality, and other agronomic traits in maize cultivars, even at the seedling stage. Currently, correlations between flavonoids and agronomic traits remain underexplored, and color-based phenotypic-assisted breeding has received limited attention. Correlations have been reported between ACNs coloration and kernel dehydration, nitrogen utilization and yield traits [41,42]. Here, we explored the potential linkage of ACNs response to LP with carbon partitioning and plant growth across maize cultivars, proving new insights into breeding selection based on tissue-specific flavonoid coloration (Figure 8). With the growing insights into flavonoid biosynthesis and its regulation, flavonoid coloration may be further exploited as a molecular marker and in phenotypic-assisted breeding, similar to its established role in crop haploid breeding [43]. Meanwhile, it is important to note that ACNs accumulation is strongly influenced by multiple environmental factors and further regulated by internal metabolites and signals beyond those discussed above (for instance, the recently reported ABI4 gene and B-box proteins) [44,45]. The complex dynamic interactions between plant development and the environment imply that seedling-stage observations alone may not reliably reflect stress tolerance across the entire growth cycle. Moreover, color-based assessments are convenient but subjective in field screening; high-efficiency and high-throughput quantitative methods remain lacking. Therefore, until more robust methodologies are established and further correlations between ACNs and agronomic traits are validated, overreliance on seedling ACNs coloration as an indicator of crop resistance to stresses should be avoided [46,47].

## 5. Conclusions

In summary, this study provides new insights into the roles of ACNs in maize cultivar evaluation under phosphorus deficiency. LP stress significantly enhanced ACNs accumulation in leaves and sheaths, with clear cultivar-specific differences distinguishing ACN-sensitive from ACN-insensitive cultivars. The ACN-sensitive cultivars exhibited consistently reduced starch accumulation but maintained higher relative growth rates under LP than those of ACN-insensitive cultivars. ACNs accumulation was positively correlated with sucrose availability and photosynthetic resilience but negatively correlated with starch reserves, underscoring its function as a carbon sink that supports stress adaptation. These findings highlight the dual role of ACNs in regulating carbon allocation and enhancing crop stress tolerance and point to their potential use as both physiological markers and breeding targets in maize improvement.

## Data Availability

The original contributions presented in this study are included in the article/Supplementary Materials. Further inquiries can be directed to the corresponding authors.

## Supplementary Materials

The following supporting information can be downloaded at: https://www.mdpi.com/article/10.3390/plants14172690/s1.

## Abbreviations

ACNs	Anthocyanins
CK	Control
FW	Fresh weight
LA	Leaf area
LP	Low phosphorus
RGR	Relative growth ratio
TSC	Total soluble sugars
DH605	Denghai605
JNK728	Jingnongke728
XY335	Xianyu335
ND108	Nongda108
WK702	Weike702
ZD958	Zhengdan958
