# Supplementary material for: Anthocyanin Synthesis Capability of Maize Cultivars Is Associated with Their Photosynthetic Carbon Partitioning for Growth Adaptability Under Low Phosphorus
Source: Plants (Basel). 2025 Aug 28;14(17):2690. doi: 10.3390/plants14172690 (PMC12429986; doi:10.3390/plants14172690)
Supplement: Supplementary file 1 [file plants-14-02690-s001.zip › plants-3803608-supplementary.pdf]

Supplementary files:

Table S1 Nutrient solution formulations under the different P treatment (CK or LP).

Name	Compound	Treatment	
		CK (mol/L)	LP (mol/L)
A	$Ca(NO_3)_2 \cdot 4H_2O$	2.0x10^{-3}	
	NH_4Cl	1.0x10^{-3}	
B	K_2SO_4	7.5x10^{-4}	
	$MgSO_4 \cdot 7H_2O$	6.5x10^{-4}	
C	KCl	1.0x10^{-4}	1.6x10^{-4}
D	KH_2PO_4	6.25x10^{-5}	2.5x10^{-6}
E	Na-Fe-EDTA	2.0x10^{-4}	
	H_3BO_3	1.0x10^{-5}	
	$MnSO_4 \cdot H_2O$	1.0x10^{-6}	
	$ZnSO_4 \cdot 7H_2O$	1.0x10^{-6}	
	$CuSO_4 \cdot 5H_2O$	5.0x10^{-7}	
	$(NH_4)_6Mo_7O_{24} \cdot 4H_2O$	5.0x10^{-5}	

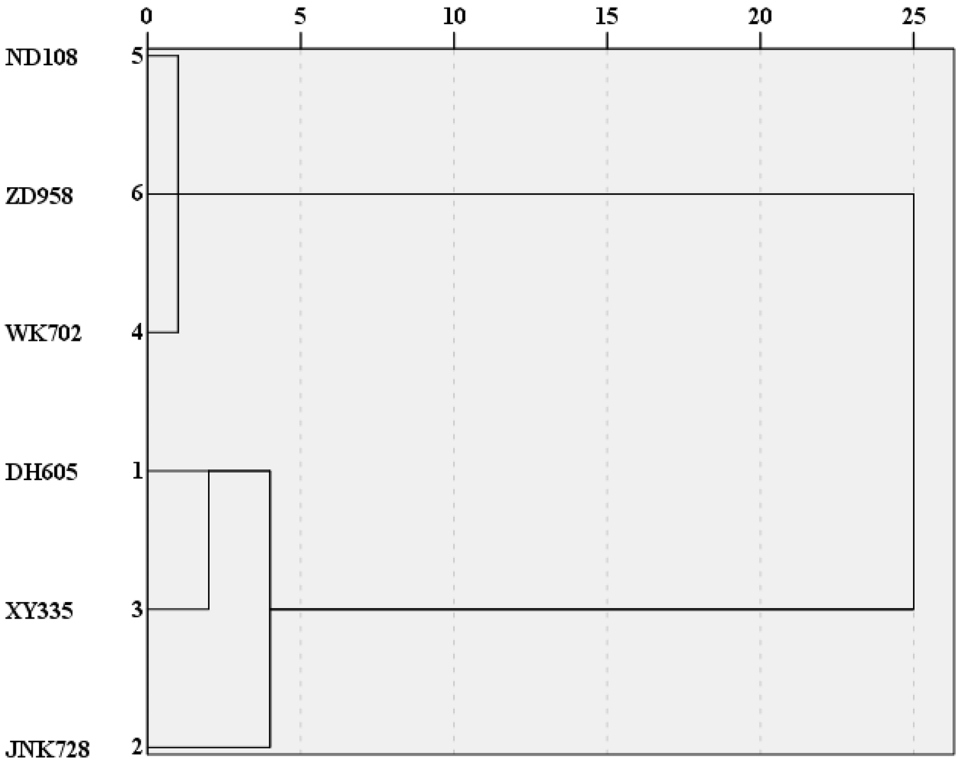

Figure S1 Cluster analysis using ward linkage by anthocyanins (ACNs) changes induced by LP in the 4th leaf and its sheath for the six cultivars. Three cultivars (WK702, ND108, ZD958) were defined as ACNs-insensitive, and the other three (DH605, JNK728, XY335) were defined as ACNs-sensitive. Abbreviations: LP, low phosphorus; DH605: Denghai605; JNK728: Jingnong728; XY335: Xianyu335; WK702: Weike702; ND108: Nongda108; ZD958: Zhengdan958.

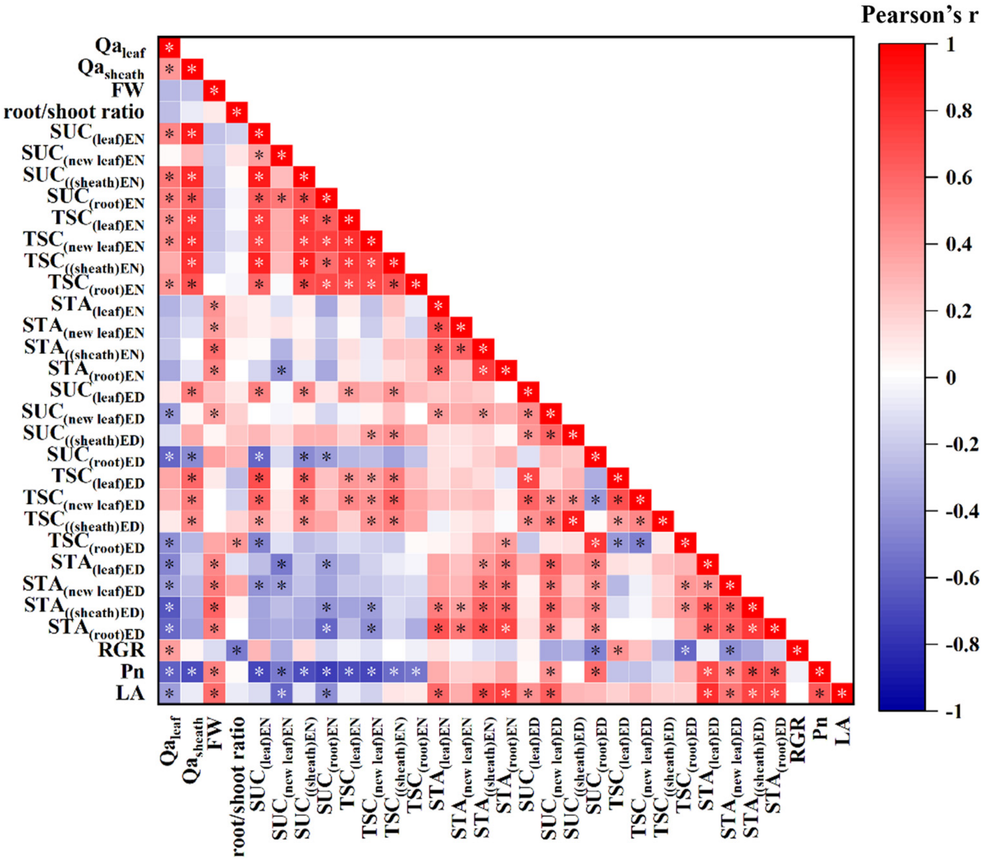

* p<=0.05

Figure S2 Correlation coefficient matrix between the anthocyanin content, the sucrose (SUC), total soluble carbohydrates (TSC) and starch (STA) levels in the 4th leaf (leaf), sheaths (sheath), unexpanded new leaves (new leaf) and roots (root) at the end of the night (EN) and at the end of the day (ED), the fresh weight (FW), the root-to-shoot ratio, the photosynthesis (Pn), the relative growth rate (RGR) and the leaf area (LA) of six cultivars under control at V4 stage. Abbreviations: Qa_{leaf}: anthocyanin content in the 4th leaf; Qa_{sheath}: anthocyanin content the sheaths of 4th leaf.

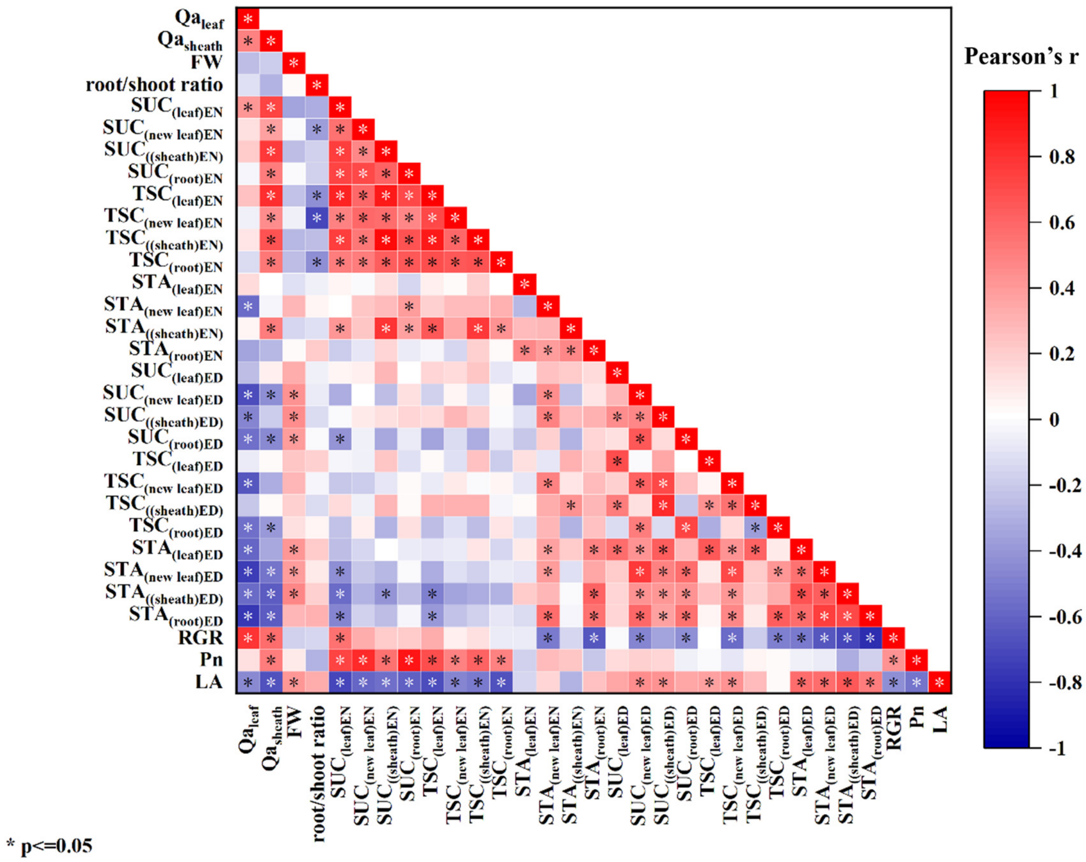

Figure S3 Correlation coefficient matrix between the anthocyanin content, the sucrose (SUC), total soluble carbohydrates (TSC) and starch (STA) content in the 4th leaf (leaf), sheaths (sheath), unexpanded new leaves (new leaf) and roots (root) at the end of night (EN) and at the end of day (ED), the fresh weight (FW), the root-to-shoot ratio, the photosynthesis (Pn), the relative growth rate (RGR) and the leaf area (LA) of six cultivars under low phosphorus at V4 stage. Abbreviations: Qa_{leaf}: anthocyanin content in the 4th leaf; Qa_{sheath}: anthocyanin content the sheaths of 4th leaf.
